# Potential of TiO_2_ with Various Au Nanoparticles for Catalyzing Mesotrione Removal from Wastewaters under Sunlight

**DOI:** 10.3390/nano10081591

**Published:** 2020-08-13

**Authors:** Daniela Šojić Merkulov, Marina Lazarević, Aleksandar Djordjevic, Máté Náfrádi, Tünde Alapi, Predrag Putnik, Zlatko Rakočević, Mirjana Novaković, Bojan Miljević, Szabolcs Bognár, Biljana Abramović

**Affiliations:** 1 Department of Chemistry, Biochemistry and Environmental Protection, Faculty of Sciences, University of Novi Sad, Trg Dositeja Obradovića 3, 21000 Novi Sad, Serbia; marina.lazarevic@dh.uns.ac.rs (M.L.); aleksandar.djordjevic@dh.uns.ac.rs (A.D.); sabolc.bognar@dh.uns.ac.rs (S.B.); biljana.abramovic@dh.uns.ac.rs (B.A.); 2 Department of Inorganic and Analytical Chemistry, University of Szeged, Dóm tér 7, H-6720 Szeged, Hungary; nafradim@chem.u-szeged.hu (M.N.); alapi@chem.u-szeged.hu (T.A.); 3 Faculty of Food Technology and Biotechnology, University of Zagreb, Pierottijeva 6, 10000 Zagreb, Croatia; 4 Institute for Nuclear Sciences “Vinča”, University of Belgrade, Mihajla Petrovića Alasa 12-14, 11351 Vinča, Belgrade, Serbia; zlatkora@vinca.rs (Z.R.); mnovakov@vinca.rs (M.N.); 5 Faculty of Technology, University of Novi Sad, Bulevar cara Lazara 1, 21000 Novi Sad, Serbia; miljevic@uns.ac.rs

**Keywords:** photocatalysis, mesotrione, TiO_2_, Au nanoparticle, scavenger, degradation intermediate

## Abstract

Nowadays, great focus is given to the contamination of surface and groundwater because of the extensive usage of pesticides in agriculture. The improvements of commercial catalyst TiO_2_ activity using different Au nanoparticles were investigated for mesotrione photocatalytic degradation under simulated sunlight. The selected system was 2.43 × 10^−3^% Au–S–CH_2_–CH_2_–OH/TiO_2_ (0.5 g/L) that was studied by transmission electron microscopy and ultraviolet-visible (UV-Vis) spectroscopy. It was found that TiO_2_ particles size was ~20 nm and ~50 nm, respectively. The Au nanoparticles were below 10 nm and were well distributed within the framework of TiO_2_. For 2.43 × 10^−3^% Au–S–CH_2_–CH_2_–OH/TiO_2_ (0.5 g/L), band gap energy was 2.45 eV. In comparison to the pure TiO_2_, addition of Au nanoparticles generally enhanced photocatalytic removal of mesotrione. By examining the degree of mineralization, it was found that 2.43 × 10^−3^% Au–S–CH_2_–CH_2_–OH/TiO_2_ (0.5 g/L) system was the most efficient for the removal of the mesotrione and intermediates. The effect of *tert*-butanol, NaF and ethylenediaminetetraacetic acid disodium salt on the transformation rate suggested that the relative contribution of various reactive species changed in following order: h^+^ > ^●^OH_ads_ > ^●^OH_bulk_. Finally, several intermediates that were formed during the photocatalytic treatment of mesotrione were identified.

## 1. Introduction

Mesotrione, or otherwise known as [2-(4-methylsulfonyl-2-nitrobenzoyl)-1,3-cyclohexanedione], is the common name for a herbicide, which controls annual broadleaf weeds in maize fields. That is the chemical isolated from the plant *Callistemon citrinus*, developed and originally marketed by Zeneca. This compound inhibits 4-hydroxyphenylpyruvate dioxygenase that is component of the biochemical pathways that converts amino acid tyrosine into molecules plastoquinone and α-tocopherol that are then used by plants [1].

Besides good properties of mesotrione for weed control, non-target organisms are exposed by additional toxic and harmful effects. Because of the low sorption of mesotrione in the soil, it has a tendency to leach to the groundwater during corn cultivation [2], where it causes negative consequences on the aquatic ecosystem [3]. In addition, toxic influence on *Tetrahymena pyriformis* nonspecific esterase activities *Vibrio fischeri* metabolism and may cause infestation of some sea life [4]. According to Du et al. [5], mesotrione and its metabolites cause algal blooms phenomena by imposing structural changes in aquatic prokaryotes. Consequently, ubiquitous use of mesotrione can become an ecological problem due to presence of its residues in the soil [6] and in the waters [7]. Generally, the removal of harmful and toxic organic pollutants from the environment presents a challenge for environmental scientists due to their effects to the surroundings. Example of effective and ecofriendly approaches for removal of organic contaminants from water is photocatalytic degradation [8,9,10,11].

There are many metal-oxides that serve as powerful photocatalysts, but the most frequently used is TiO_2_ [9,12,13,14,15]. This compound has good features like biological and chemical stability, availability, insolubility in water, acids and bases, resistance to photocorrosion, low cost, and nontoxicity [12,16,17]. Unfortunately, TiO_2_ has a large band gap (e.g. 3.0–3.2 eV) for formation of electron-hole (e^−^–h^+^) pairs, which limits its application in the visible part of the spectrum. Another drawback of TiO_2_ is the fast recombination of photogenerated e^−^–h^+^ pairs that negatively affects the efficiency of photocatalytic degradation. One of the ways for improving the photocatalytic activity of TiO_2_ in the visible part of the spectrum is alteration with metals like Cu, Ni, Co, Mn, Cr, Ru, Fe, Pt, Ag, and Au [9]. Recently, great attention was given to Au nanoparticles since its coupling with TiO_2_ has showed extended spectral response in the visible region of light [18,19,20] with efficient retardation of e^–^–h^+^ recombination [21,22]. Reported results [18,19,20] have confirmed that enhancement of TiO_2_ with Au nanoparticles in the visible part of the spectrum is due to surface plasmon resonance, i.e., collective oscillation of free conduction band electrons. Here, Au nanoparticles were able to absorb photons and form excited electrons under visible light irradiation. Moreover, electrons can be additionally shifted to the TiO_2_ conduction band, while positive holes stay on the metal nanoparticles [23]. Surface modification of quantum dots is achieved by adding capping or functionalized agents. Here, with addition of the chemical agents, surface of nanoparticles can alter the particle size, morphology, mechanical stability, optical properties, toxicity, and photocatalytic activities [24].

Thiol-stabilized gold nanoparticles have gained increased attention because of their catalytic potential, nanoelectronics, optics, as well as chemical and biological sensing and biomedicine [25,26,27,28,29,30,31]. Initially, thiol groups were used for stabilizing gold nanoparticles, however this technique has been adapted to prepare Au nanoparticles of ultrasmall size (<2 nm). Furthermore, due to atomic packing mode in ultrasmall metal nanoparticles (clusters), different optical and electronic properties were exhibited, as compared to the larger gold nanoparticles. Gold clusters have tendency to lose metallic nature due to quantum confinement effect, while the collective plasmon excitation is no longer supported. Moreover, clusters exhibit highest-occupied molecular orbital (HOMO) and lowest-unoccupied molecular orbital (LUMO) electronic properties and step-wise optical absorption behavior [32]. Besides that, investigators have been reported functionalization of fullerenes with metal nanoparticles in order to achieve novel materials with unique optoelectronic and catalytic properties [33,34,35]. The functionalization can be achieved by reaction of gold nanoparticles with mercapto derivatives of fullerene [34,36,37] or by reactions between fullerene and gold protected with amine moieties on the surfaces [33,38].

Considering that numerous authors have reported enhanced efficiency of photocatalytic degradation using modified catalysts with Au nanoparticles, this study investigated whether or not the improvement of catalyst may be achieved by different *n*/*n* (%) of Au nanoparticles and suspension of commercially available catalyst TiO_2_. Nanoparticles of Au (Au) and modified Au with: 2-mercaptoethanol (Au–S–CH_2_–CH_2_–OH), as well as Au–S–CH_2_–CH_2_–OH modified with fullerenol nanoparticles (Au–S–CH_2_–CH_2_–OH–FNP) were tested for the mesotrione photocatalytic degradation efficiency with TiO_2_ and simulated sunlight. Characterization, degree of mineralization and study of the selected systems was evaluated in detail. This was additional to the assessment of heterogeneous catalysis efficiency and different effects of scavengers. Finally, identification of intermediates was performed for indicated reaction mechanism and to confirm the role(s) of ^●^OH and/or direct charge transfer reactions during the transformation process.

## 2. Materials and Methods

### 2.1. Chemicals, Solutions and Catalysts

All chemicals were of reagent grade and were used without further purification. Mesotrione (CAS No 104206-82-8, C_14_H_13_NO_7_S, *M*_r_ = 339.32, PESTANAL^®^, analytical standard, 99.9% purity) was purchased from Fluka (Buchs, Switzerland); 85% H_3_PO_4_ and 35% HCl were obtained from Lachema (Neratovice, Czech Republic); 99.8% acetonitrile (ACN) and *tert*-butanol, 99.9% from Sigma-Aldrich (St. Louis, MO, USA); ethylenediaminetetraacetic acid disodium salt (EDTA × 2Na), Dojindo (Rockville, MD, USA); colloidal gold (EAN: 4313042704413, Vitalpur Berlin, Germany, ~0.03 g/L); ≥99.0% 2-mercaptoethanol (Sigma-Aldrich); 99–100% formic acid, VWR (Darmstadt, Germany) and NaF, Kemika (Zagreb, Croatia). All solutions were made using ultrapure water. TiO_2_ alone (Sigma-Aldrich, anatase, surface area 35–65 m^2^/g), and in combination with Au, Au–S–CH_2_–CH_2_–OH and Au–S–CH_2_–CH_2_–OH–FNP were used as photocatalyst.

### 2.2. Synthesis of Au–S–CH_2_–CH_2_–OH and Au–S–CH_2_–CH_2_–OH–FNP Nanoparticles

The volume of 12 mL of Au nanoparticle solution (concentration ~0.03 g/L) was intensively stirred (750 rpm) at +4 °C for 30 min. Then 0.026 mL of HS–CH_2_–CH_2_–OH at +4 °C was added. Reaction mixture intensely stirred 48 h in dark, while the synthesis of fullerenol C_60_(OH)_24_ nanoparticles was previously described [39,40]. In 5 mL of Au–S–CH_2_–CH_2_–OH nanoparticles, 0.05 mL FNP (concentration 0.0125 g/L) was added and sonicated for 15 min. The solution was left to rest for 12 h in the dark at 23 °C.

### 2.3. Characterization of Nanoparticles

Powder TiO_2_ samples were dispersed in distilled water/ethanol and the suspension was treated in ultrasound for 5 min. A drop of very dilute suspension was placed on a holey-carbon-coated copper grid and dried by evaporation at ambient temperature.

Transmission electron microscopy (TEM), high-resolution transmission electron microscopy (HRTEM), and scanning transmission electron microscopy (STEM) were performed on a FEI Talos F200X microscope (Thermo Fisher Scientific, Waltham, MA, USA) operating at 200 keV. Images were recorded on a CCD camera with resolution of 4096 × 4224 pixels using the ‘User interface software package.’ An energy dispersive X-ray spectroscopy (EDX) system attached to the TEM operating in the STEM mode was used to analyze the chemical composition of the samples. High-angle annular dark-field (HAADF) image presented in the paper was captured in nanoprobe-TEM mode with a camera length of ~200 mm. All of the presented digital images were analyzed, but not processed.

The absorption coefficient of the light (*α*) of the newly synthesized nanocomposite was measured by ultraviolet-visible (UV-Vis) spectrophotometer Evolution 600 (Thermo Fisher Scientific, Waltham, MA, USA), in the electromagnetic spectrum range between 240 nm and 840 nm with the step of 1 nm and speed of 10 nm/min. Demineralized water was used as a reference during the measurements.

### 2.4. Photodegradation Procedure

The photocatalytic degradation was carried out in a cell using halogen lamp with detailed characteristics described in the Supplementary Material. The experiments were carried out using 20 mL of mesotrione solution (0.05 mM) containing different molar ratios *n*/*n* (%) of Au nanoparticles and 10 mg or 40 mg of catalyst TiO_2_ depending on the experiment. Experimental procedure for the mesotrione photocatalytic degradation was described in the Supplementary Material. All experiments were performed at the pH of ~4. In the investigation of the influence of *^●^*OH/h^+^ scavenger, *tert*-butanol, NaF or EDTA × 2Na were added to the reaction mixture.

### 2.5. Analytical Procedure

For the kinetic studies of the mesotrione photocatalytic degradation, samples of the reaction mixture were taken before the beginning the experiments (0 min of irradiation) and at different time intervals during irradiation (volume variation ca. 10%). The suspensions were filtered through membrane filters (Millex-GV, Millipore, MA, USA, 0.22 µm) in order to separate the catalyst particles. The preliminary check confirmed the absence of mesotrione adsorption on the filters. After that, a 20 µL of the sample was injected and analyzed in the UFLC (Shimadzu, Tokyo, Japan) with UV-Vis diode-array detector (DAD) (wavelength of mesotrione maximum absorption at 225 nm) and column Inertsil^®^ ODS-4 (GL Sciences, Eindhoven, the Netherlands, 50 mm × 2.1 mm, particle size 2 µm). When recording the chromatogram, an isocratic elution with a mobile phase consisted of 39.5% ACN and 60.5% aqueous solution of 0.1% H_3_PO_4_ (flow rate: 0.38 mL/min) was used. For the calibration of the instrument for analysis of mesotrione, standard solutions with concentration range from the 0.0002 to 0.10 mM were prepared by dilution of the stock solution. Concentrations of mesotrione in different time intervals of irradiation have been calculated by the appropriate peak areas and linear equations obtained by the linear regression of a calibration curve. Correlation coefficient for the calibration curve was 0.999.

Changes in the pH during the degradation were monitored by using a combined glass electrode (pH-Electrode SenTix 20, WTW, Thermo Fisher Scientific, MA, USA) connected to the pH-meter (pH/Cond 340i, WTW). In order to determine mineralization degree, total organic carbon (TOC) analysis was done. Aliquots of 10 mL of the reaction suspension were taken before the beginning the experiments (0 min of irradiation) and after 180 min of irradiation (each separate probe is performed). After that, aliquots diluted to 25 mL and analyzed on a Liqui TOC II analyzer (Elementar, Langenselbold, Germany) according to Standard US 120 EPA Method 9060A.

For the high-performance liquid chromatography–mass spectrometry (HPLC/MS) evaluation of intermediates increasingly concentrated solution of mesotrione was treated (0.1 mM). The assays of the samples prepared for HPLC/MS analysis were performed by Agilent 1100 HPLC. Kinetex column (XB-C18 100 A, pore size 2.6 µm; Phenomenex Inc., Torrance, CA, USA) was the stationary phase and the mobile phase consisted of 30% ACN and 70% aqueous solution of 0.1% formic acid (flow rate: 0.75 mL/min). Agilent 1100 HPLC was coupled with a DAD and LC/MSD VL mass spectrometer (Agilent Technologies, Santa Clara, CA, USA) equipped with Electrospray Ionization (ESI), source and a triple quadrupole analyzer (QqQ). DAD at various wavelengths (210 nm, 230 nm, 260 nm, 290 nm) was used. Both positive and negative ionization modes were used to optimize the MS parameters, and all compounds were detected with higher sensitivity in the negative mode. Consequently, the deprotonated molecule-ion ([M–H]^−^) and its fragments were detected and all *m*/*z* values reported in study are related to the deprotonated forms.

## 3. Results and Discussion

### 3.1. Characterization

**Figure 1:**
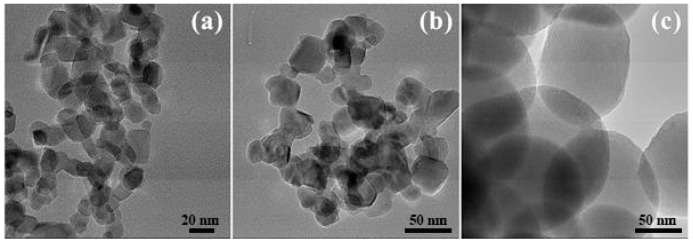
Low-magnification TEM bright-field images of 2.43 × 10^−3^% Au–S–CH_2_–CH_2_–OH/TiO_2_ (0.5 g/L) nanocomposites: irregular shaped (**a**,**b**) and spherical shaped (**c**) particles.

TEM was used to investigate particle size, crystallinity and morphology of 2.43 × 10^−3^% Au–S–CH_2_–CH_2_–OH/TiO_2_ (0.5 g/L) nanocomposites. Figure 1a–c shows low magnification bright-field images of the 2.43 × 10^−3^% Au–S–CH_2_–CH_2_–OH/TiO_2_ (0.5 g/L) sample, taken at different areas and with typical morphology. As it can be seen, the TiO_2_ particles have irregular (Figure 1a,b) or spherical (Figure 1c) shapes. Furthermore, it can be estimated that the size of the particles with the irregular morphology was within the range of 10 to 40 nm, with most of them with diameter of approximately 20 nm. The spherical TiO_2_ particles were larger and with the diameters above 50 nm.

**Figure 2:**
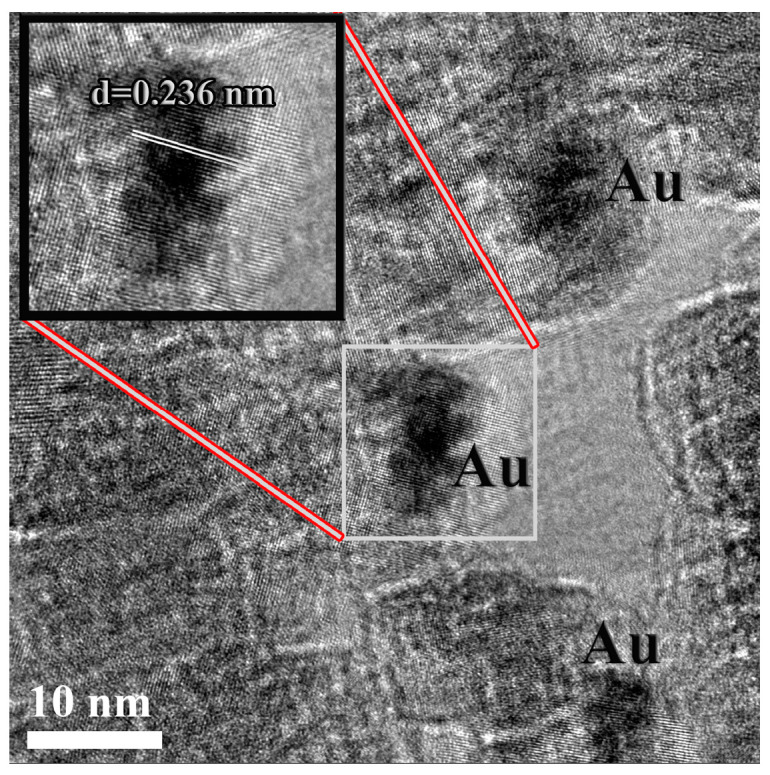
HRTEM image of 2.43 × 10^−3^% Au–S–CH_2_–CH_2_–OH/TiO_2_ (0.5 g/L) sample representing Au nanoparticles distributed over the TiO_2_ matrix. Inset has enlarged section of selected Au nanoparticles with the marked crystalline planes.

The structures of the TiO_2_ and 2.43 × 10^−3^% Au–S–CH_2_–CH_2_–OH/TiO_2_ (0.5 g/L) samples were further analyzed at high magnifications and a typical HRTEM image (Figure 2). It was observed that the Au nanoparticles were below 10 nm in size and were well distributed within the TiO_2_ framework. The nanoparticles were easily distinguished based on the image contrast, as being darker in the contrast and compared to the surrounding matrix. The TiO_2_ and Au nanoparticles framework was highly crystalline, as evidenced from the well resolved crystalline lattices. The enlarged section of the selected area, presenting Au nanoparticles with the marked crystalline planes was given in the inset. We estimated the interplanar spacing of ~0.236 nm, which was in a good agreement with the known value for Au (111) of 0.2355 nm [41].

**Figure 3:**
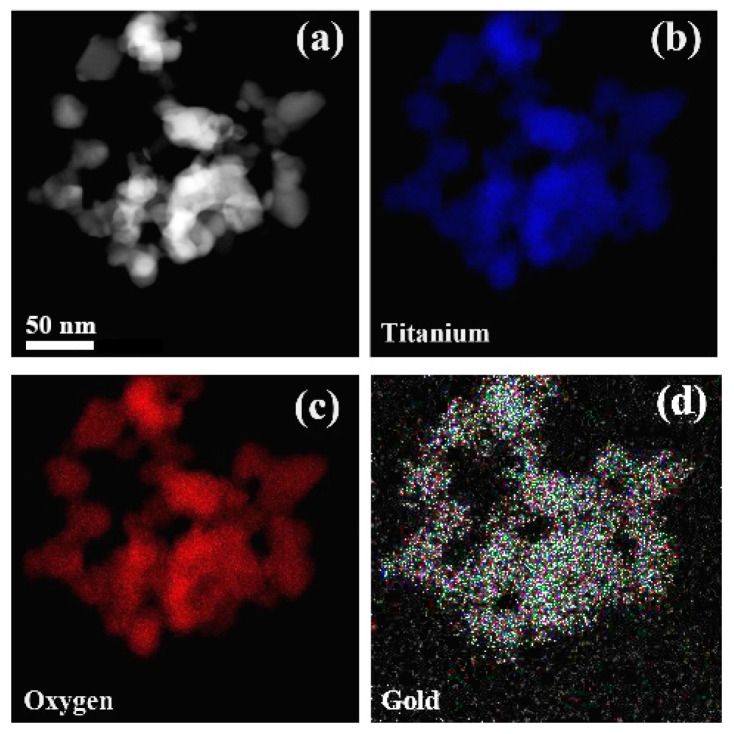
STEM–HAADF image (**a**) and low-magnification elemental mapping images of 2.43 × 10^−3^% Au–S–CH_2_–CH_2_–OH/TiO_2_ (0.5 g/L) sample (**b**–**d**). The elements were distinguished by the color: titanium (blue), oxygen (red) and gold (green).

Further insights into the chemical nature of the 2.43 × 10^−3^% Au–S–CH_2_–CH_2_–OH/TiO_2_ (0.5 g/L) samples were provided by using STEM–EDX measurements. Figure 3 presents STEM–HAADF image (Figure 3a) and corresponding elemental mapping (Figure 3b–d) taken at the sample area presented in Figure 1b. Different elements’ elemental color mapping was used, wherein the titanium was labeled as blue, oxygen as red and gold atoms as green color. The images showed uniform spatial distribution of gold over the TiO_2_ particles, confirming the Au was well incorporated into the TiO_2_ matrix.

Figure S1 shows the absorbance *α* in the function of the wavelength of the illuminated light in the samples. The sample 2.43 × 10^−3^% Au–S–CH_2_–CH_2_–OH/TiO_2_ (0.5 g/L) exhibited an absorption function similar to the pure TiO_2_, while the pure Au–S–CH_2_–CH_2_–OH possessed an absorption drop-off in the UV region.

**Figure 4:**
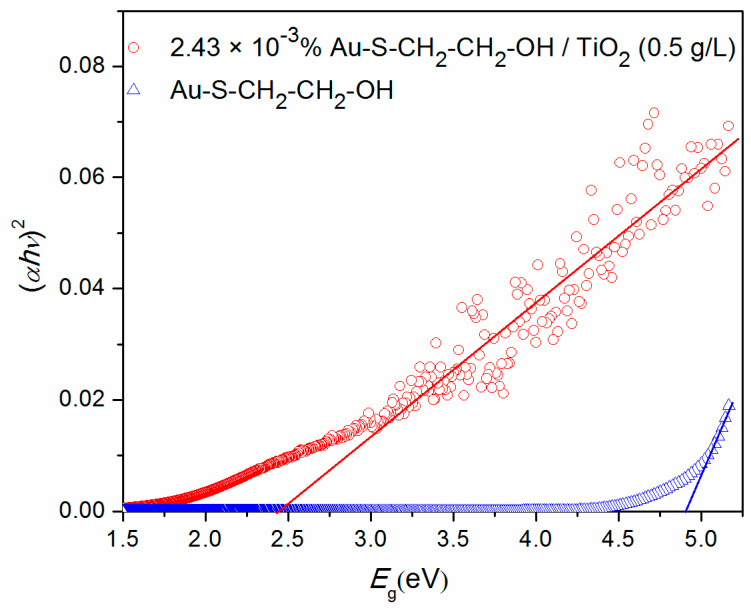
Modified absorbance ${\left(\alpha h\nu \right)}^{2}$ plotted vs. the energy *hν* for the samples 2.43 × 10^−3^% Au–S–CH_2_–CH_2_–OH/TiO_2_ (0.5 g/L) (red circles) and Au–S–CH_2_–CH_2_–OH (blue triangles). The colored lines are the linear extrapolations show the band gap energies.

The band gap energies (*E*_g_) of the samples were obtained using the Tauc’s plot [42], that was based on the fact that *α* is dependent on the *E*_g_ of the absorbing material (Kubelka-Munk theory) [43,44]. The *E*_g_ can be determined from a plot of the modified absorbance ${\left(\alpha h\nu \right)}^{n}$ vs. the energy *hν* by extrapolating the linear fit of the straight section to the *α* = 0 intercept of the energy coordinate (Figure 4). The factor *n* depends on the transition type and it was assumed to be a direct allowed (*n* = 2).

The band gap energies, calculated from the experimental data as described above, were shown in the Table S2. For the sample 2.43 × 10^−3^% Au–S–CH_2_–CH_2_–OH/TiO_2_ (0.5 g/L), the *E*_g_ of 2.45 eV belongs to the visible part of the electromagnetic spectrum, corresponding to the light of 506 nm. This result was similar to the one of the pure TiO_2_, which has the *E*_g_ of 2.55 eV, and corresponding to the light of 486 nm [45]. The literature band gap value for pure titania (3.0–3.2 eV) corresponds to a bulk and this shift could be attributed to the quantum confinement effect as TiO_2_ used in this study has a much smaller average crystal size of approximately 6 nm and much larger specific surface area as compared to the commonly used TiO_2_ P25 [46,47]. On the other hand, pure Au–S–CH_2_–CH_2_–OH has very high band gap energy of 4.90 eV, corresponding to the UV light of 253 nm. Regardless of the fact that Au is a metal, a sample made of Au nanoparticles modified with 2-mercaptoethanol (Au–S–CH_2_–CH_2_–OH) was measured as solution which is according to the results, a wide band-gap semiconductor. In addition, the Au nanoparticles solution is of rather low concentration (0.03 g/L) and, therefore, there was not much influence to the solution’s electric properties expected.

### 3.2. Photolytic and Photocatalytic Degradation

**Figure 5:**
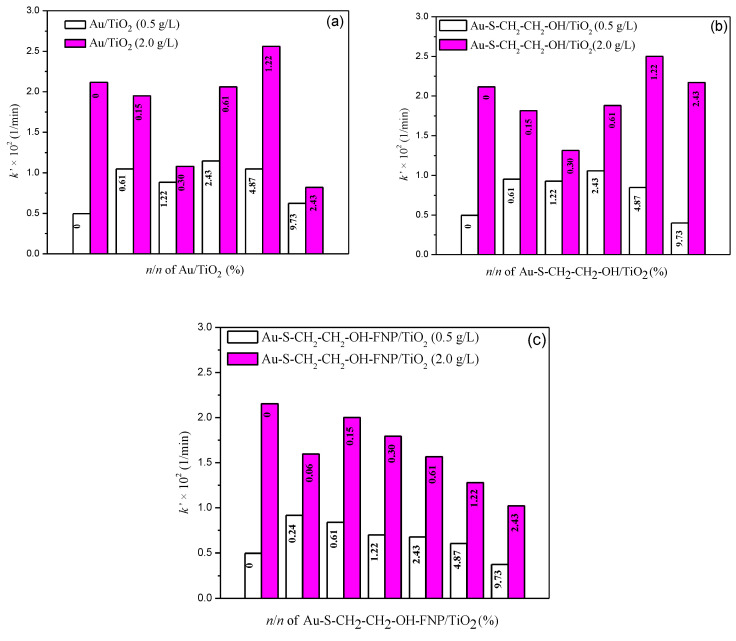
The influence of different *n*/*n* × 10^3^ (%) of: (**a**) Au; (**b**) Au–S–CH_2_–CH_2_–OH; and (**c**) Au–S–CH_2_–CH_2_–OH–FNP/TiO_2_ (0.5 g/L and 2.0 g/L) on the *k*′ determined for the first 120 min of mesotrione (0.05 mM) photocatalytic degradation under simulated sunlight.

Considering that Au nanoparticles have the potential to enhance removal of organic pollutants, mesotrione photolytic and photocatalytic degradation combined with different *n*/*n* (%) of Au, Au–S–CH_2_–CH_2_–OH, as well as Au–S–CH_2_–CH_2_–OH–FNP in the absence/presence of TiO_2_ at two loading levels (0.5 and 2.0 g/L) were investigated. In the presence of different Au nanoparticles, the degradation of mesotrione was negligible under simulated sunlight (Figure S2). Further, in the presence of pure TiO_2_ the rate of transformation constants increased with loading level and was found to be 0.496 × 10^−2^ 1/min (*r* = 0.992) and 2.115 × 10^−2^ 1/min (*r* = 0.997) after 120 min of irradiation (Figure 5). It was found in the literature that the rate of photocatalytic degradation increases with catalyst loading as a consequence of the increasing the number of active sites in the solution [48].

All kinetic curves shown in Figures S3 and S4 in the first 120 min of irradiation could be fitted reasonably well by an exponential decay curve suggesting the pseudo-first kinetics order using the following Equation (1):

ln(*c*_o_/*c*) = *k’t* (1)

where *c* is the mesotrione concentration, *c*_o_ the initial concentration of mesotrione, *t* the time of irradiation, and *k*′ apparent first-order rate constant.

#### 3.2.1. Activity of TiO_2_ (0.5 g/L) without and with Different Au Nanoparticles

Obtained results for the influence of different *n*/*n* (%) of Au, Au–S–CH_2_–CH_2_–OH or Au–S–CH_2_–CH_2_–OH–FNP and TiO_2_ on the efficiency of mesotrione photocatalytic degradation were presented in Figure 5. Findings showed that for different *n*/*n* (%), addition of Au enhanced the photocatalytic degradation of mesotrione, as compared to the TiO_2_ alone (Figure 5a). The highest progression in efficiency of the photocatalytic degradation of mesotrione was observed at 2.43 × 10^−3^% Au/TiO_2_ (0.5 g/L). However, further enhancements of up to 9.73 × 10^−3^% decreased the efficiency of mesotrione removal. Regarding the most efficient system, 2.43 × 10^−3^%, 89% of herbicide was removed after 180 min of irradiation. Furthermore, system without Au for the same irradiation time showed only 59% of mesotrione removal (Figure S3).

Gold nanoparticles were modified with 2-mercaptoethanol with the intention to investigate influence of functionalization agents on the efficiency of mesotrione photocatalytic degradation with TiO_2_ by using simulated sunlight (Figure 5b). Similar as with the case of Au, addition of different *n*/*n* (%) Au–S–CH_2_–CH_2_–OH/TiO_2_ (0.5 g/L) of up to 2.43 × 10^−3^% resulted in enhanced efficiency of mesotrione removal. This was in contrast to further additions where efficiency of removal decreased. Namely, the optimal *n*/*n (*%*)* of Au–S–CH_2_–CH_2_–OH/TiO_2_ (0.5 g/L) has proved to be 2.43 × 10^−3^%, when 87% of herbicide was removed after 180 min of irradiation (Figure S3).

In our previous work [45], fullerenol improved the efficiency of TiO_2_ where we synthesized a molecule of Au–S–CH_2_–CH_2_–OH with fullerenol nanoparticles attached. In the case of 0.24 × 10^–3^% Au–S–CH_2_–CH_2_–OH–FNP/TiO_2_ (0.5 g/L) (Figure 5c) the best improvement was achieved, wherein 79% of mesotrione was removed after 180 min of irradiation (Figure S3). With the increase of *n*/*n* (%), the efficiency of herbicide photocatalytic degradation decreased.

As previously mentioned, the reason for better catalytic performances of 2.43 × 10^−3^% Au/TiO_2_ (0.5 g/L) might be the band gap energy, as in that case it shifted towards the lower values, hence there was efficacious use of visible light in relation to the TiO_2_ or Au–S–CH_2_–CH_2_–OH.

#### 3.2.2. Activity of TiO_2_ (2.0 g/L) with/without Different Au Nanoparticles

Similar as before, Au nanoparticles were investigated for the effect on the mesotrione photocatalytic degradation with loading of 2.0 g/L TiO_2_ under simulated sunlight (Figure 5 and Figure S4). From the obtained results, it can be seen that only 1.22 × 10^−3^% Au/TiO_2_ (2.0 g/L) and 2.43 × 10^−3^% Au/TiO_2_ (2.0 g/L) systems had influence on efficiency of mesotrione removal, as compared to the TiO_2_ alone where both, increase and decrease was noticed. The influence of different *n*/*n* (%) of Au–S–CH_2_–CH_2_–OH/TiO_2_ (2.0 g/L) was also investigated, where better mesotrione photocatalytic degradation efficiency was noticed in the case of 1.22 × 10^−3^% vs. TiO_2_ alone. Different *n*/*n* (%) of Au–S–CH_2_–CH_2_–OH–FNP/TiO_2_ (2.0 g/L) either decreased or had no influence on mesotrione photocatalytic degradation.

### 3.3. Evaluation of Mineralization

**Figure 6:**
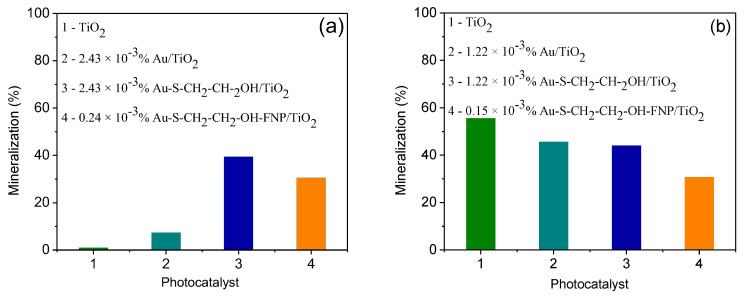
Mineralization of mesotrione (0.05 mM) after 180 min of photocatalytic degradation under simulated sunlight with different Au nanoparticles and TiO_2_: (**a**) 0.5 g/L and (**b**) 2.0 g/L.

In order to estimate the quality of water after photocatalytic degradation, mineralization of mesotrione was determined for the best systems with/without different Au nanoparticles, at both TiO_2_ loading levels. From the obtained results, for the case of TiO_2_ loading of 0.5 g/L (Figure 6a) without Au nanoparticles there was no mineralization observed, while addition of different Au increased the percentage of mineralization. The highest percentage of mineralization showed the system 2.43 × 10^−3^% Au–S–CH_2_–CH_2_–OH/TiO_2_ (0.5 g/L), where 39.5% of organic matter was mineralized. In the case of higher TiO_2_ loading (2.0 g/L), addition of Au nanoparticles decreased the percentages of mineralization (Figure 6b). Moreover, addition of Au–S–CH_2_–CH_2_–OH and Au–S–CH_2_–CH_2_–OH–FNP showed no improvements of mineralization at 2.0 g/L vs. 0.5 g/L TiO_2_. Hence, it may be concluded that the addition of Au–S–CH_2_–CH_2_–OH or Au–S–CH_2_–CH_2_–OH–FNP to TiO_2_ suspension at loading of 0.5 g/L improved degree of mineralization that was similar to the corresponding systems at 2.0 g/L loading of TiO_2_.

### 3.4. Effect of Hydroxyl Radicals and Holes Scavengers

With the purpose to evaluate reactive species involved in the reaction kinetics of mesotrione photodegradation with 2.43 × 10^−3^% Au–S–CH_2_–CH_2_–OH/TiO_2_ (0.5 g/L), ^●^OH and h^+^ scavengers were added to the reaction mixtures. Furthermore, the roles of ^●^OH can be estimated through addition of different alcohols or NaF. Namely, addition of *tert*-butanol (*k*(*tert*-butanol + ^●^OH) = 6.00 × 10^8^ L/(mol s) [49]), revealed how goes the extended reaction through bulk ^●^OH (^●^OH_bulk_), because of the low affinity for TiO_2_ surfaces. However, F^−^ showed strong adsorption on TiO_2_ surfaces, so NaF can scavenge adsorbed ^●^OH (^●^OH_ads_) [50]. Additionally, EDTA × 2Na was used for scavenging of photogenerated h^+^ [51] and ^●^OH (*k*(EDTA + ^●^OH) = 4.00 × 10^8^ L/(mol s) [49]). EDTA was well adsorbed on the TiO_2_ surfaces, thus reacts primarily with ^●^OH_ads_. Besides this, EDTA reacts with photogenerated h^+^ via direct charge transfers, which is highly enhanced by the adsorption due to the interactions of Ti≡OH and carboxyl groups of EDTA.

**Figure 7:**
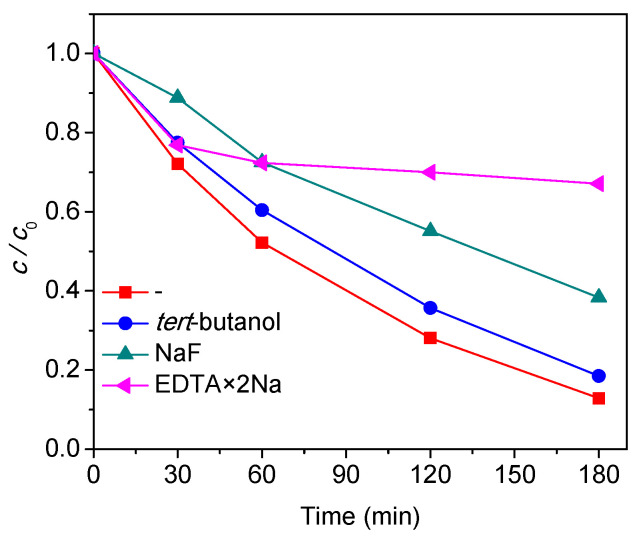
Effects of h^+^ and ^●^OH scavengers (10 mM) on the efficiency of mesotrione (0.05 mM) photocatalytic degradation in the presence of 2.43 × 10^−3^% Au–S–CH_2_–CH_2_–OH/TiO_2_ (0.5 g/L) under simulated sunlight.

From the obtained results (Figure 7), it can be seen that 10 mM NaF mainly inhibited the degradation efficiency of mesotrione in the first 30 min of irradiation, where in the case of 10 mM *tert*-butanol and 10 mM EDTA × 2Na there was no significant inhibitions. Based on this, it can be concluded that photocatalytic degradation of mesotrione took place via ^●^OH_ads_ during the first 30 min of irradiation. After initial period of mesotrione photodegradation EDTA × 2Na had better inhibition vs. the addition of the NaF (the rate constant is 5.33 × 10^–3^ 1/min (*r* = 0.998) after 180 min of irradiation). Finčur et al. [51] used EDTA as a scavenger of h^+^, and according to their findings, it can be concluded that h^+^ had significant roles in photocatalytic degradation of alprazolam by TiO_2_ Degussa P25.

Further, the effect of F^–^ on the clomazone degradation efficiency in TiO_2_ suspension was investigated [50]. The results showed that the degradation rate remained the same with the addition of F^–^ of up to 8.0 mM NaF. In the presence of 8.0 mM NaF the degradation rate slightly decreased, while in the presence of *tert*-butanol slight reduction of efficacy for mesotrione photocatalytic degradation was observed during the 180 min of irradiation with the rate constant of 9.30 × 10^–3^ 1/min (*r* = 0.998). Here the absence of any scavenger yielded rate constant of 11.26 × 10^–3^ 1/min (*r* = 0.999). This phenomenon can be consequence of acidic conditions, where additional to ^●^OH, other active species take parts in photocatalytic degradation of a target compound, as photo-generated h^+^ and *tert*-butanol could not inhibit the reaction to the expected extent [52]. This was in agreement with our results. Namely, after 30 min of irradiation, the main path of degradation was through h^+^ and less through ^●^OH_ads_, while ^●^OH_bulk_ had low influence.

### 3.5. LC–ESI–MS Identification of Mesotrione Degradation Intermediates

**Figure 8:**
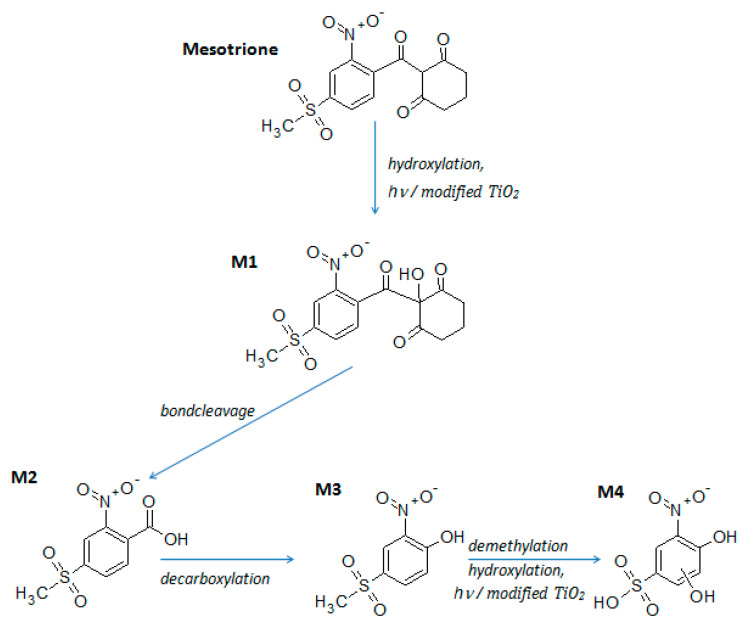
Mesotrione and its stable products detected during the process of photocatalytic degradation in the presence of 2.43 × 10^−3^% Au–S–CH_2_–CH_2_–OH/TiO_2_ (0.5 g/L) under simulated sunlight.

The formation of stable products during the photocatalytic treatment of mesotrione in the presence of 2.43 × 10^−3^% Au–S–CH_2_–CH_2_–OH/TiO_2_ (0.5 g/L) was analyzed using HPLC/MS with ESI ionization in the negative mode (Table 1). Mesotrione was detected as a deprotonated anion ([M-H]^‒^) at *m*/*z* = 338.2 Da. The first step of the transformation was the most likely the addition of ^●^OH (Figure 8), which resulted with the formation of M1 product (*m*/*z* = 354.3). Jović et al. [53] also reported the formation of a similar product as the first stable species. Further transformations of M1 via bond cleavage leads to the formation of product M2 (*m*/*z* = 244.2). Its fragment detected at *m*/*z* = 200.2 is probably formed due to the decarboxylation from M2 (Figure 8). Thus M2 is most likely the 4-(methanesulfonyl)-2-nitrobenzoic acid, a natural metabolite of mesotrione [54,55], which has been detected as the primary product in the case of various advanced oxidative processes [53,56]. The stable product M3 (*m*/*z* = 216.2) is the 4-(methanesulfonyl)-2-nitrophenol, which is formed via decarboxylation from product M2 [53]. Product M4 (*m*/*z* 234.2) is probably formed from product M3 via demethylation and addition of another ^●^OH to the aromatic ring (Figure 8).

The MS spectrum of detected products is presented in Figure S5. Although *tert*-butanol has no significant effect on the transformation rate, detected products proved that hydroxylation has important roles in the transformation. It should be noted that the formation of hydroxylated products is possible by direct charge transfer, and not only by ^●^OH-initiated transformation.

## 4. Conclusions

The results of this study clearly indicated that the photocatalytic treatment using TiO_2_ (0.5 and 2.0 g/L) modified with Au, Au–S–CH_2_–CH_2_–OH, as well as Au–S–CH_2_–CH_2_–OH–FNP nanoparticles can efficiently eliminate mesotrione under simulated sunlight from water. The reaction followed the pseudo-first order kinetics. The addition of all types of Au nanoparticles to the suspension of TiO_2_ (0.5 g/L) in different *n*/*n* (%) enhanced the degradation efficacy of mesotrione, as compared to the TiO_2_ alone. Contrary to this, the efficiency of degradation decreased or had no impacts in the most cases with addition of different Au nanoparticles in TiO_2_ (2.0 g/L) suspension. On the basis of TOC measurements, the degree of mineralization in water was mostly improved at 2.43 × 10^−3^% Au–S–CH_2_–CH_2_–OH/TiO_2_ (0.5 g/L). This system was identified as the most efficient in the photocatalytic degradation of mesotrione and further was characterized by TEM and UV-Vis spectroscopy techniques. It was found that TiO_2_ particles had irregular or spherical shapes with their respective sizes of ~20 nm or above 50 nm. Besides, Au nanoparticles were below 10 nm and were well distributed within the framework of TiO_2_. The *E*_b_ for the system 2.43 × 10^−3^% Au–S–CH_2_–CH_2_–OH/TiO_2_ (0.5 g/L) was 2.45 eV and belonged to the visible part of the electromagnetic spectrum, while pure TiO_2_ had *E*_b_ of 2.55 eV for the same range. Furthermore, the presence of *tert*-butanol, NaF and EDTA × 2Na caused the inhibition of photocatalytic degradation of mesotrione in the following order h^+^ > ^●^OH_ads_ > ^●^OH_bulk_. Several degradation intermediates were formed and identified by the LC–ESI–MS technique. Further investigations should be focused on the development of new nanomaterials and their applications for the photocatalytic degradations of organic pollutants from wastewaters at industrial level.

## Figures and Tables

**Table 1:** Retention time of chromatography peak, detected *m*/*z* values (the first is the precursor ion, the fragments are listed below with relative abundance), and the calculated molecular mass of the mesotrione and the detected intermediates. (*m*/*z* value is related to the deprotonated form ([M–H]^‒^, while *M* is the average mass of molecule calculated by the ChemSketch program).

Peak label	*t*_r_ (min)	*m*/*z* ([M–H]^‒^)	*M* (Da)
Mesotrione	6.10	338.2 (100), 291.2 (34), 339.2 (17)	339.3
M1	2.61	354.3 (100), 113.2 (93), 97.1 (25)	355.3
M2	1.75	244.2 (100), 200.2 (86), 62.2 (28)	245.2
M3	2.81	216.2 (100), 213.2 (45), 91.2 (30), 212.3 (9)	217.2
M4	2.10	234.2 (100), 91.2 (50), 157.1 (48), 214.2 (41), 62.2 (21)	235.2

## Supplementary Materials

The following are available online at https://www.mdpi.com/2079-4991/10/8/1591/s1, photodegradation and analytical procedure details, Figure S1: The measured *α* of the samples: 2.43 × 10^–3^% Au–S–CH_2_–CH_2_–OH/TiO_2_ (0.5 g/L) (red squares), TiO_2_ (black triangles) and Au-S-CH_2_-CH_2_-OH (blue circles), Table S1: The band gap energies and their corresponding wavelengths, Figure S2: The influence of different *n* × 10^8^ (mol) of Au nanoparticles on mesotrione (0.05 mM) photolytic degradation using simulated sunlight, Figure S3: The influence of different *n*/*n* × 10^3^ (%) of: (a) Au; (b) Au–S–CH_2_–CH_2_–OH, as well as (c) Au–S–CH_2_–CH_2_–OH–FNP and TiO_2_ (0.5 g/L) on the efficiency of mesotrione (0.05 mM) photocatalytic degradation under simulated sunlight, Figure S4: The influence of different *n*/*n* × 10^3^ (%) of: (a) Au; (b) Au–S–CH_2_–CH_2_–OH, as well as (c) Au–S–CH_2_–CH_2_–OH–FNP and TiO_2_ (2.0 g/L) on the efficiency of mesotrione (0.05 mM) photocatalytic degradation under simulated sunlight, Figure S5: The MS spectra of mesotrione and detected products (ESI, negative mode).
