# Supplementary material for: Potential of TiO2 with Various Au Nanoparticles for Catalyzing Mesotrione Removal from Wastewaters under Sunlight
Source: Nanomaterials (Basel). 2020 Aug 13;10(8):1591. doi: 10.3390/nano10081591 (PMC7466515; doi:10.3390/nano10081591)
Supplement: Supplementary file 1 [file nanomaterials-10-01591-s001.pdf]

Supplementary Material

Potential of TiO_2 with Various Au Nanoparticles for Catalyzing Mesotrione Removal from Wastewaters under the Sunlight

Daniela Šojić Merkulov [1,*], Marina Lazarević [1], Aleksandar Djordjevic [1], Máté Náfrádi [2], Tünde Alapi [2], Predrag Putnik [3,*], Zlatko Rakočević [4], Mirjana Novaković [4], Bojan Miljević [5], Szabolcs Bognár [1] and Biljana Abramović [1]

[1] Department of Chemistry, Biochemistry and Environmental Protection, Faculty of Sciences, University of Novi Sad, Trg Dositeja Obradovića 3, 21000 Novi Sad, Serbia; marina.lazarevic@dh.uns.ac.rs (M.L.); aleksandar.djordjevic@dh.uns.ac.rs (A.Dj.); sabolc.bognar@dh.uns.ac.rs (S.B.); biljana.abramovic@dh.uns.ac.rs (B.A.)

[2] Department of Inorganic and Analytical Chemistry, University of Szeged, H-6720 Szeged, Dóm tér 7, Hungary; nafradim@chem.u-szeged.hu (M.N.); alapi@chem.u-szeged.hu (T.A.)

[3] Faculty of Food Technology and Biotechnology, University of Zagreb, Pierottijeva 6, 10000 Zagreb, Croatia

[4] Institute for Nuclear Sciences "Vinča", University of Belgrade, Mihajla Petrovića Alasa 12-14, 11351 Vinča, Belgrade, Serbia; zlatkora@vinca.rs (Z.R.); mnovakov@vinca.rs (M.N.)

[5] Faculty of Technology, University of Novi Sad, Bulevar cara Lazara 1, 21000 Novi Sad, Serbia; miljevic@uns.ac.rs

* Correspondence: daniela.sojic@dh.uns.ac.rs (D.Š.M.); pputnik@alumni.uconn.edu (P.P.)

Material and Methods

Photodegradation procedure

Photocatalytic degradation was carried out in a cell made of Pyrex glass (total volume of ca. 40 mL, liquid layer thickness 35 mm), with a plain window on which the light beam was focused. The cell was equipped with a magnetic stirring bar and water circulating jacket. Irradiation in the visible spectral range was performed using a 50 W halogen lamp (Philips). Energy fluxes of radiation were measured using Delta Ohm (Padova, Italy) radiometer with sensors: LP 471 UVA (spectral region 315–400 nm) for the UVA region and LP 471 RAD (spectral region 400–1050 nm) for the Vis region. The photon flux for the halogen lamp was 63.85 MW/cm^2 for visible radiation and 0.22 MW/cm^2 for the UVA region.

The experiments were carried out using 20 mL of 0.05 mM solution of mesotrione containing 10 or 40 mg of TiO_2 (except for the study of photolysis). Different volume of gold nanoparticles (Au or Au–S–CH_2–CH_2–OH or Au–S–CH_2–CH_2–OH–FNP) was added in the suspension depending on experiment. Thus obtained suspension was sonicated in the dark for 15 min before irradiation in order to achieve adsorption-desorption equilibrium and uniform particle size of catalyst, and at the same time thermostatted at 25.0 °C at a constant stream of O_2 (3.0 mL/min). Adsorption of mesotrione on the surface of photocatalysts was checked every time after 15 min of suspension sonication in the dark (before irradiation) wherein adsorption was not established (only ±1–2% that was within the limits of the measurement error). Then suspension was irradiated and stirred at a constant speed, whereby streaming of O_2 was continued.

Results and Discussion

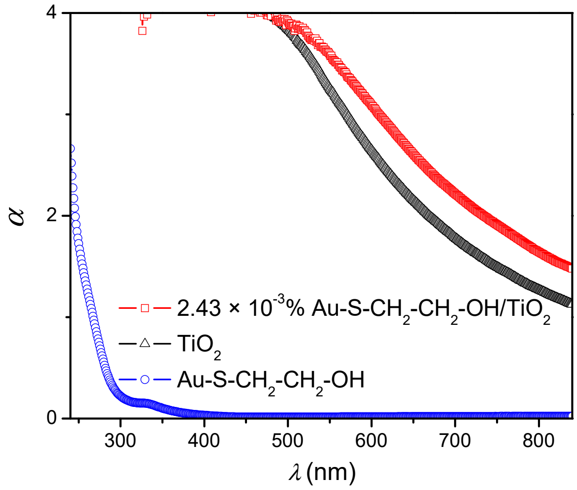

Figure S1. The measured α of the samples: 2.43 × 10^{-3}% Au–S–CH_2–CH_2–OH/TiO_2 (0.5 g/L) (red squares), TiO_2 (black triangles) and Au–S–CH_2–CH_2–OH (blue circles).

Table S1. The band gap energies and their corresponding wavelengths.

Sample	E_g (eV)	λ (nm)
2.43 × 10^{-3}% Au–S–CH_2–CH_2–OH/TiO_2 (0.5g/L)	2.45	506
TiO_2	2.55	486
Au–S–CH_2–CH_2–OH	4.90	253

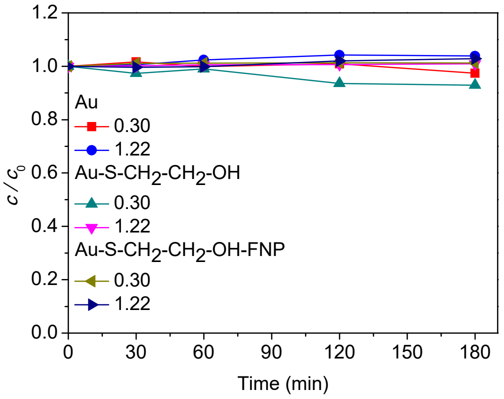

Figure S2. The influence of different $n \times 10^8$ (mol) of Au nanoparticles on mesotrione (0.05 mM) photolytic degradation using simulated sunlight.

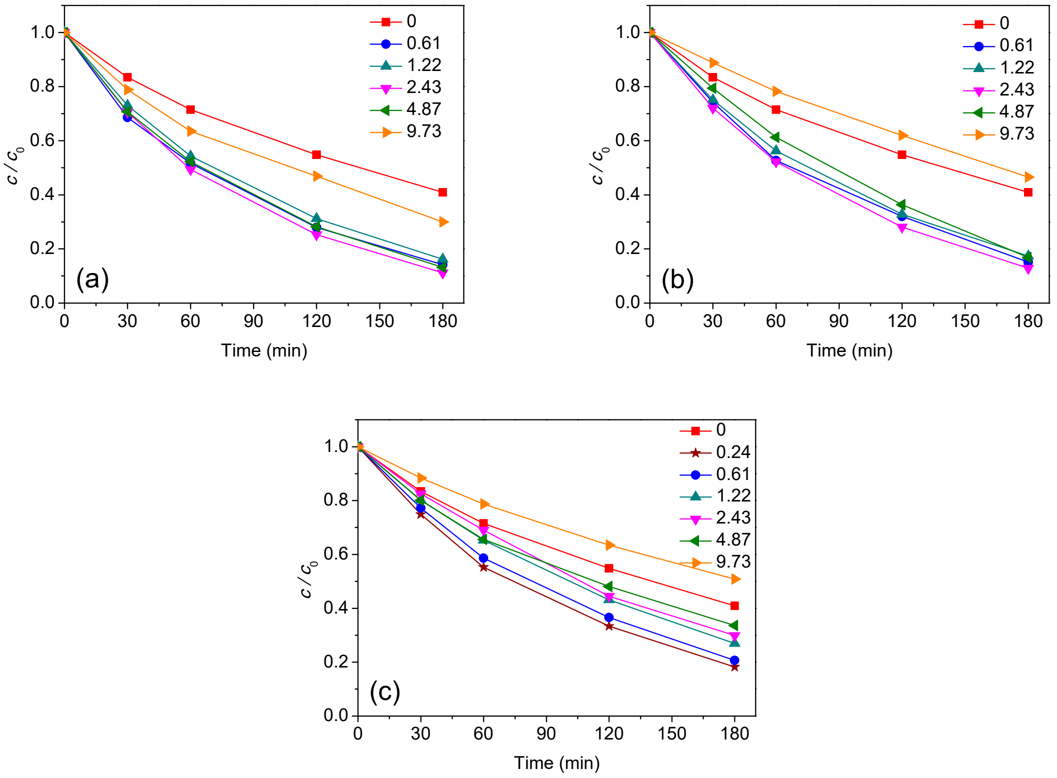

Figure S3. The influence of different *n*/*n* × 10^3 (%) of: (**a**) Au; (**b**) Au–S–CH_2–CH_2–OH, as well as (**c**) Au–S–CH_2–CH_2–OH–FNP and TiO_2 (0.5 g/L) on the efficiency of mesotrione (0.05 mM) photocatalytic degradation under simulated sunlight.

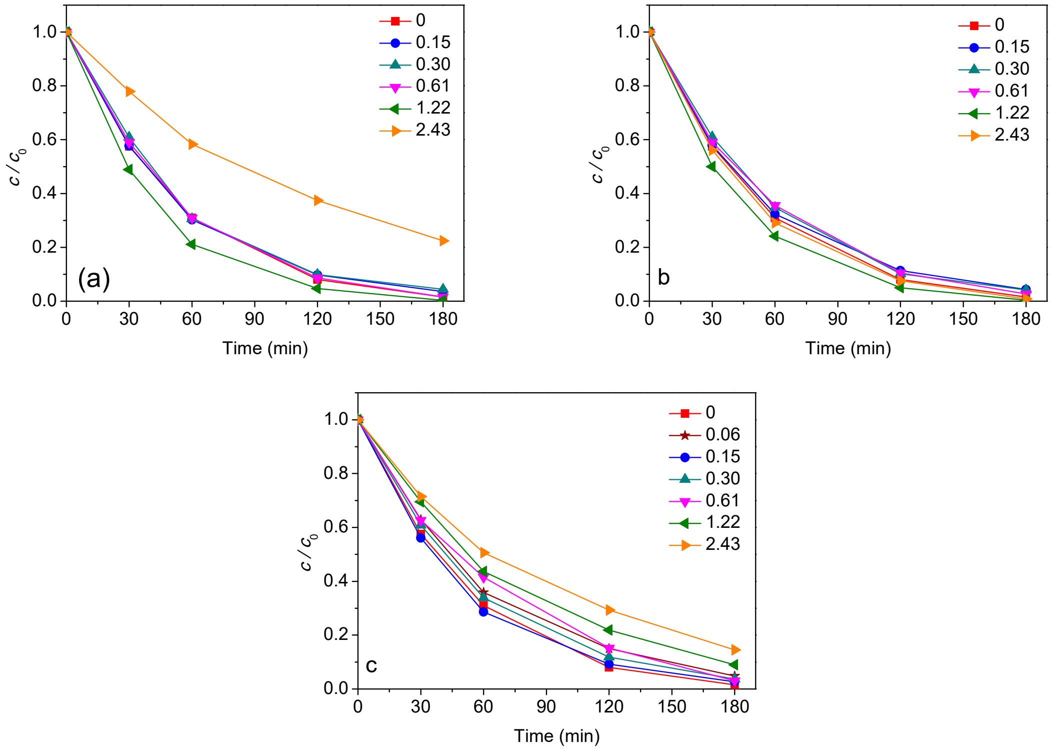

Figure S4. The influence of different *n*/*n* × 10^3 (%) of: (**a**) Au; (**b**) Au–S–CH_2–CH_2–OH, as well as (**c**) Au–S–CH_2–CH_2–OH–FNP and TiO_2 (2.0 g/L) on the efficiency of mesotrione (0.05 mM) photocatalytic degradation under simulated sunlight.

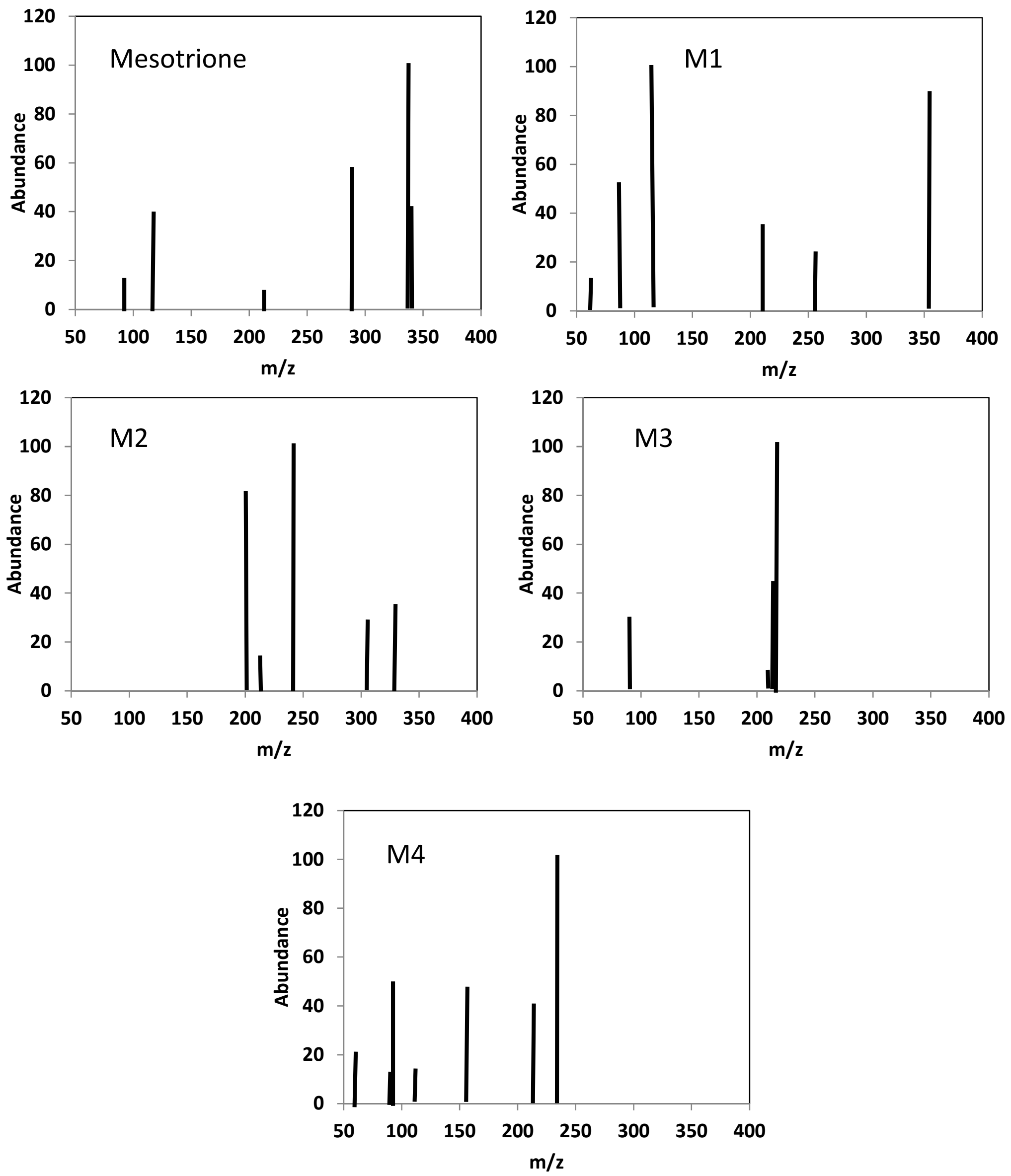

Figure S5. The MS spectra of mesotrione and detected products (ESI, negative mode).
